# Fasting regulates EGR1 and protects from glucose- and dexamethasone-dependent sensitization to chemotherapy

**DOI:** 10.1371/journal.pbio.2001951

**Published:** 2017-03-30

**Authors:** Stefano Di Biase, Hong Seok Shim, Kyung Hwa Kim, Manlio Vinciguerra, Francesca Rappa, Min Wei, Sebastian Brandhorst, Francesco Cappello, Hamed Mirzaei, Changhan Lee, Valter D. Longo

**Affiliations:** 1 Longevity Institute, Leonard Davis School of Gerontology and Department of Biological Sciences, University of Southern California, Los Angeles, California, United States of America; 2 Institute for Liver and Digestive Health, Royal Free Hospital, University College London (UCL), London, United Kingdom; 3 Center for Translational Medicine (CTM), International Clinical Research Center (ICRC), St. Anne's University Hospital, Brno, Czech Republic; 4 Centro Studi Fegato (CSF)-Liver Research Center, Fondazione Italiana Fegato, Trieste, Italy; 5 Euro-Mediterranean Institute of Science and Technology, Palermo, Italy; 6 Department of Experimental Biomedicine and Clinical Neuroscience, University of Palermo, Palermo, Italy; 7 IFOM, FIRC Institute of Molecular Oncology, Milano, Italy; Duke University, United States of America

## Abstract

Fasting reduces glucose levels and protects mice against chemotoxicity, yet drugs that promote hyperglycemia are widely used in cancer treatment. Here, we show that dexamethasone (Dexa) and rapamycin (Rapa), commonly administered to cancer patients, elevate glucose and sensitize cardiomyocytes and mice to the cancer drug doxorubicin (DXR). Such toxicity can be reversed by reducing circulating glucose levels by fasting or insulin. Furthermore, glucose injections alone reversed the fasting-dependent protection against DXR in mice, indicating that elevated glucose mediates, at least in part, the sensitizing effects of rapamycin and dexamethasone. In yeast, glucose activates protein kinase A (PKA) to accelerate aging by inhibiting transcription factors Msn2/4. Here, we show that fasting or glucose restriction (GR) regulate PKA and AMP-activated protein kinase (AMPK) to protect against DXR in part by activating the mammalian Msn2/4 ortholog early growth response protein 1 (EGR1). Increased expression of the EGR1-regulated cardioprotective peptides atrial natriuretic peptide (ANP) and B-type natriuretic peptide (BNP) in heart tissue may also contribute to DXR resistance. Our findings suggest the existence of a glucose–PKA pathway that inactivates conserved zinc finger stress-resistance transcription factors to sensitize cells to toxins conserved from yeast to mammals. Our findings also describe a toxic role for drugs widely used in cancer treatment that promote hyperglycemia and identify dietary interventions that reverse these effects.

## Author summary

Fasting can selectively protect normal cells against the deleterious side effects of chemotherapy while sensitizing cancer cells to therapy. This is, in part, due to the reallocation of energy from growth to protection in normal cells but not cancer cells in response to reduced nutrient availability. Because glucose sensitizes yeast cells to stress and considering that several widely used drugs that increase glucose levels are administered in combination with chemotherapy, we tested the role of glucose reduction on the sensitivity of normal cells and mice to the chemotherapy drug doxorubicin (DXR). We show that fasting or glucose restriction (GR) reduce protein kinase A (PKA) activation and increase AMP-activated protein kinase (AMPK) activity. These signal transduction changes cause the activation of the conserved zinc finger stress-resistance transcription factor early growth response protein 1 (EGR1) (Msn2/4 in yeast) to protect cardiomyocytes from doxorubicin toxicity. These results provide evidence for a glucose-sensing pathway that negatively regulates a stress response conserved from yeast to mammalian cells. These studies also suggest that the common use of drugs that cause hyperglycemia, such as dexamethasone (Dexa) and rapamycin, could increase the toxicity of cancer drugs to normal tissues and organs while making glucose available to cancer cells.

## Introduction

Despite advances in cancer therapy, the standard of care predominantly includes chemotherapy, radiotherapy, or their combination. These treatments are associated with a multitude of side effects ranging from discomfort to the development of secondary tumors and severe toxicity to multiple systems [1–4]. Doxorubicin (DXR) is an antineoplastic drug widely used against a variety of human malignancies, including bladder, breast, leukemia, lung, liver, and ovarian cancer, but its clinical use is largely limited by dose-dependent cardiac toxicity [5, 6]. To increase the efficacy of cancer treatment or to help with the management of the adverse effects of the therapy, other drugs such as dexamethasone (Dexa), aprepitant, and lorazepam are often used in combination with chemo- and radiotherapy [7, 8]. The corticosteroid Dexa is mainly used as a palliative drug but can also be effective in the treatment of multiple myeloma, leukemia, and lymphoma [9–12]. However, treatment with Dexa can raise blood glucose levels [13, 14], and several studies have proposed that it can promote toxicity in the brain [15, 16].

Multiple studies have shown the antitumor and antimitotic effect of the mTOR (mammalian target of rapamycin) inhibitor rapamycin (Rapa) in different model organisms [17]. Multiple analogs of Rapa (temsirolimus and everolimus) have been approved by the FDA for the clinical treatment of subependymal giant cell astrocytoma (SEGA), advanced hormone receptor–positive HER2-negative breast cancer, primitive neuroectodermal tumors (PNETs) of pancreatic origin, and for advanced renal cell carcinoma (RCC) [18, 19] (see also the National Institutes of Health-National Cancer Institute website). Despite its efficacy in reducing the progression of certain tumors, mTOR inhibitors also promote hyperglycemia, raising the possibility that, similarly to Dexa, they could also increase chemotherapy toxicity to normal tissues [20, 21].

Fasting or short-term starvation (STS) and fasting-mimicking diets (FMDs) are effective in improving both the life span and health span of multiple species [22–24]. STS can also selectively protect normal (but not cancer) cells against toxins including chemotherapy, a phenomenon we named differential stress resistance (DSR), but the cellular mechanisms responsible for its protective effects are unclear [22, 25–29].

We have previously shown that yeast Msn2/4 [27, 30] are stress response transcription factors negatively regulated by glucose and protein kinase A (PKA) that mediate part of the fasting-dependent effects on stress resistance and life span extension [25, 29]. Mammalian early growth response protein 1 (EGR1), which has high homology to yeast Msn2/4, is a key transcription factor involved in diverse cellular processes such as development, proliferation, DNA repair, apoptosis, and tumor suppression [31–33]. In mammals, *Egr1* expression is also up-regulated by the master energy sensor AMP-activated protein kinase (AMPK) [9, 34, 35], which is downstream of and negatively regulated by PKA [36–38]. However, the role of mammalian EGR1 in cellular protection and its link to glucose and PKA/AMPK has not been investigated.

Here, we tested the hypothesis that fasting reverses the hyperglycemic effect of Dexa and Rapa to protect against DXR-induced cardiotoxicity [28, 39] by regulating a novel mammalian glucose–PKA–AMPK–EGR1 pathway, analogous to the glucose–PKA–AMPK–Msn2/4 pathway, which regulates aging and stress resistance in yeast.

## Results

### Short-term starvation protects from DXR-induced cardiotoxicity

We have previously shown that STS effectively reduces the mortality of mice exposed to otherwise lethal doses of DXR (DSR) [28, 39]. To investigate whether the protective effects of STS involve reduction of DXR-induced cardiotoxicity, C57BL/6 mice were administered multiple doses of DXR ± STS (48 h; S1A Fig). The dose and schedule of treatment were chosen to provide prolonged exposure to DXR through multiple cycles to model the clinical doses and schedules.

**Fig 1:**
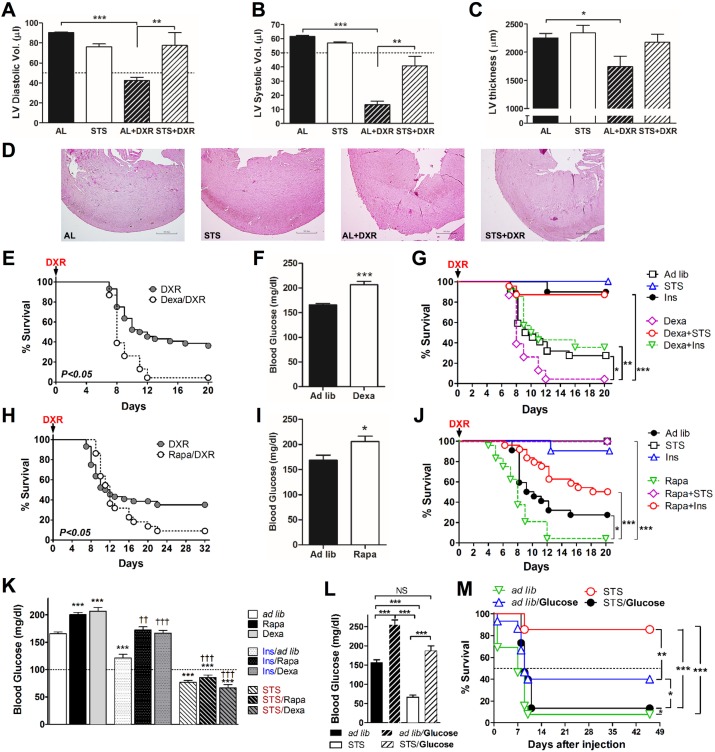
Short-term starvation (STS) and circulating-glucose reduction protect against doxorubicin (DXR)-induced cardiotoxicity. **(A, B)** Echocardiographic and **(C, D)** histological analyses were performed following multiple DXR injections in order to assess the heart function. **(A)** Diastolic and **(B)** systolic left ventricle (LV) volume (*n* = 4 to 7) and LV wall thinning **(C)** (*n* = 6 to 9) of mice undergoing ad lib (AL), STS, DXR, and STS + DXR treatments are shown. Mice pretreated with dexamethasone (Dexa) (*n* = 24) or rapamycin (Rapa) (*n* = 22) were also injected with a high-dose DXR (24 mg/kg); survival and blood glucose levels (before DXR administration) are shown in E–F and H–I, respectively. **(G, J)** STS and insulin administration increased resistance against DXR toxicity and **(K)** reduced Dexa- and Rapa-induced hyperglycemia. **(L)** Mice were administrated glucose every 4 h for 24 h prior to DXR injection (24 mg/kg/mouse; intravenous [IV]) to directly increase blood glucose levels. Glucose levels were measured 1 h after the last glucose injection, at the same time of DXR administration. **(M)** Glucose-induced hyperglycemia prevented STS-induced protection (differential stress resistance [DSR]). Experiments were repeated twice. Data represent mean ± standard error of the mean (SEM). ANOVA (and Tukey post-analysis) was performed in A, B, C, K, and L; Student’s *t* test was performed in F and I; and log-rank test was performed on E, G, H, J, and M. *p*-values < 0.05 are considered significant (*p*-value < 0.05, 0.01, and 0.001 are indicated as *, *, and ***, respectively). Underlying data and method of statistical analysis are provided in S1 Data.

Compared to the control group, DXR-treated mice displayed a reduction in cardiac function and/or functional markers, including diastolic (*p*-value < 0.0001) and systolic (*p*-value < 0.0001) left ventricle (LV) volume and wall thickness (*p*-value < 0.05) (Fig 1A–1D). STS prior to DXR injection improved the overall response to high-dose chemotherapy, prevented the loss of LV diastolic and/or systolic volumes (*p*-value < 0.001 and < 0.0001, respectively) (Fig 1A and 1B), and ameliorated LV wall thinning (Fig 1C and 1D).

### Hyperglycemia induced by Rapa and Dexa increases cardiotoxicity

STS down-regulates signal transduction proteins, including mTOR and PKA, and reduces circulating IGF-1 and glucose levels [25, 28, 39]. Because glucose is a key sensitizer of yeast cells, we tested the role of glucose in animals undergoing DXR treatment. Hyperglycemia was induced in C57BL/6 mice by daily injections with Dexa or Rapa—two agents that cause hyperglycemia and insulin resistance [40, 41] and are FDA-approved for use in cancer therapy [42, 43]—for 2 wk before DXR treatment (Fig 1E–1J and S1B Fig). Both Dexa and Rapa treatment enhanced the toxicity of DXR in mice (*p*-value < 0.05) (Fig 1G and 1J). To examine whether the reduced survival was caused by elevated circulating glucose levels, mice pretreated with Dexa and Rapa were also administered with either insulin or underwent STS in order to reduce blood glucose levels (Fig 1K). STS or insulin administration following Rapa and Dexa prevented hyperglycemia and protected mice from DXR toxicity (Fig 1G, 1J and 1K), suggesting that the hyperglycemia induced by Dexa or Rapa is responsible for the sensitization of mice to DXR-induced chemotoxicity. Notably, STS was more potent in preventing this hyperglycemia-induced sensitization than insulin treatment alone (*p*-value < 0.0001), indicating that fasting has a broader effect that is partly independent of glucose. Further, restoring hyperglycemia during STS by direct glucose injections (2g/kg IP every 4 h during the 24 h prior to DXR administration, determined based on its clearance rate [44]), restored euglycemia and fully reversed the STS-dependent protection from DXR in mice (Fig 1L and 1M). Because both high levels of insulin (injections) and low levels of insulin (caused by STS) were associated with protection against DXR, these results indicate that reduced glucose and not altered insulin levels are responsible for STS-induced protection.

### Glucose restriction protects cardiomyocytes through the PKA/EGR1 pathway

**Fig 2:**
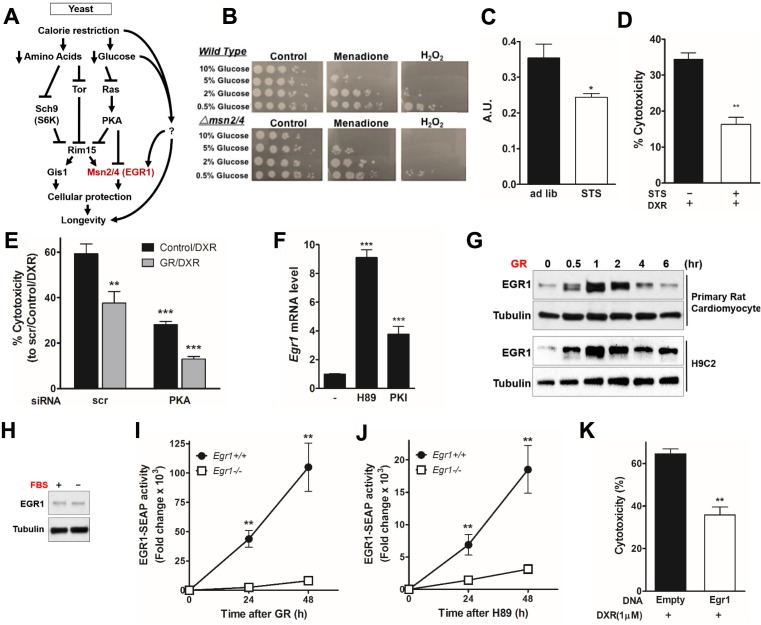
Glucose restriction (GR) induces early growth response protein 1 (*Egr1*) expression through protein kinase A (PKA)- and AMP-activated protein kinase (AMPK)-dependent regulation in cardiomyocytes. **(A)** In yeast, nutrient-sensing pathways controlled by Sch9, Tor, and Ras/PKA converge on the downstream protein kinase Rim15. Transcription factors Msn2/4 and Gis1 enhance cellular protection through transactivation of stress response genes, leading to life span extension. **(B)** Four-day-old yeast cultured in different concentrations of glucose and treated with KPO_4_ buffer only, 200μM menadione, or 100mM H_2_O_2_ and incubated for 1 h before spotting in a 10-fold serial dilution on yeast extract peptone dextrose (YPD) plates. Spot test analysis reveals a heightened sensitivity to oxidative stress as glucose concentration increases. In addition, treatment with menadione and H_2_O_2_ resulted in a 5–10-fold increase in sensitivity in the *Δmsn2/4* deletion background. **(C)** PKA kinase activity in cardiomyocytes was reduced in vitro by short-term starvation (STS) medium conditions for 48 h (*n* = 6) A.U.: arbitrary units. **(D)** Effect of STS on doxorubicin (DXR)-induced cardiotoxicity. Primary rat cardiomyocytes were incubated in STS or normal medium for 24 h and then treated with DXR (1 μM) for additional 24 h. Cytotoxicity was assessed by lactate dehydrogenase (LDH) release. **(E)** Cardiomyocytes were transfected with short interfering RNA (siRNA) against *PKA* (or a nonspecific control) at day 1, treated with GR or normal medium at day 2, and treated with DXR (1 μM) at day 3. Cytotoxicity is reported. **(F)** PKA inhibition triggers *Egr1* expression. Primary rat cardiomyocytes were treated with PKA inhibitors, H89, or PKI for 1 h. mRNA expression level of *Egr1* was measured. **(G)** Primary and H9c2 rat cardiomyocytes were incubated with GR medium for the indicated time and immunoblotted with an anti-EGR1 antibody. (**H**) Primary rat cardiomyocytes were incubated with serum-free medium for 1 h and immunoblotted with an anti-EGR1 antibody. **(I, J)** *Egr1*^*+/+*^ and *Egr1*^*-/-*^ mouse embryonic fibroblasts (MEFs) transiently transfected with EGR1-secreted alkaline phosphatase (SEAP) reporter were incubated with **(I)** GR or **(J)** H89. SEAP activity was measured and quantified by using a chemiluminescent assay. **(K)** H9c2 cardiomyocytes transfected with a Flag-*Egr1* expression vector were treated with DXR (1 μM) for 24 h. Cytotoxicity was measured by LDH release. Experiments were repeated three times, and the average for each technical repeat is reported in each graph (*n* = 3). The significance of the differences between experimental groups was determined by using one-way ANOVA (Tukey post-analysis test). Comparisons between groups were performed with Student’s *t* test. Data represent the mean ± SEM. *p*-values < 0.05 were considered significant (*p*-value < 0.05, 0.01, and 0.001 are indicated as *, *, and ***, respectively). Underlying data and method of statistical analysis are provided in S1 Data.

The reduction of PKA signaling during fasting promotes anti-aging and stress resistance in yeast and mammalian cells [23, 29, 45]. In yeast, glucose activates PKA, which in turn sensitizes cells in part by down-regulating stress resistance transcription factors Msn2/4. We therefore tested the involvement of these genes in mediating the protective effect of STS and glucose restriction (GR) (Fig 2A) [29]. GR protected yeast from oxidative stress, and the deletion of Msn2/4 reduced such resistance by 10-fold (Fig 2B). Consistent with the yeast data, in vitro STS conditions with reduced glucose (0.5 g/L) and growth factors (1% fetal bovine serum [FBS]) reduced PKA activity (*p*-value < 0.05) (Fig 2C) and protected cardiomyocytes against DXR toxicity (*p*-value < 0.001) (Fig 2D). PKA knockdown was achieved by using short interfering RNA (siRNA) against *Pkaα* (also called *Prkaca*), the main catalytic subunit of PKA [46]. PKA knockdown in H9C2 rat cardiomyocytes significantly protected from DXR toxicity, which was further improved by glucose restriction (GR) (*p*-value < 0.0001) (Fig 2E). GR also increased levels of *Egr1* transcript (S2A Fig), as did treatment with the selective PKA inhibitors H-89 and PKI 14–22 amide (PKI) (Fig 2F). GR, but not serum restriction (FBS), rapidly induced *Egr1* expression in cardiomyocytes, indicating that *Egr1* expression is regulated by glucose levels and is not strongly affected by growth and other factors contained in the serum (Fig 2G and 2H).

In order to test the action of EGR1 in response to starvation in a different cell type, we examined the effects of GR and PKA inhibition using wild-type and *Egr1*-null mouse embryonic fibroblasts (MEFs). Similar to our observation in primary cardiomyocytes, EGR1 protein levels in wild-type MEFs were rapidly induced by GR (S2E Fig). To examine whether starvation regulates the transcriptional activity of *Egr1*, we generated an *Egr1*-dependent secreted alkaline phosphatase (SEAP) reporter construct containing two copies of an *Egr1* binding element. Wild-type and *Egr1*-null MEFs were transfected with *Egr1*-SEAP constructs and treated with GR medium. A 48-h GR induced and accumulated *Egr1*-dependent SEAP expression (*p*-value < 0.001; Fig 2I). Similarly, the inhibition of PKA using H-89 increased *Egr1* activity (*p*-value < 0.05; Fig 2J). Conversely, the over-expression of *Egr1* alone protected cardiomyocytes from DXR (Fig 2K). Interestingly, PKA inhibition with H-89 was five times less potent than GR in activating *Egr1*, suggesting that GR can also promote *Egr1*-dependent transcription activity through pathways independent of PKA. These results indicate that STS and GR protect cells against DXR stress by activating *Egr1* by both PKA-dependent and independent mechanisms.

### Fasting induces the expression of cardioprotective peptide hormones ANP and BNP in an EGR1-dependent manner

**Fig 3:**
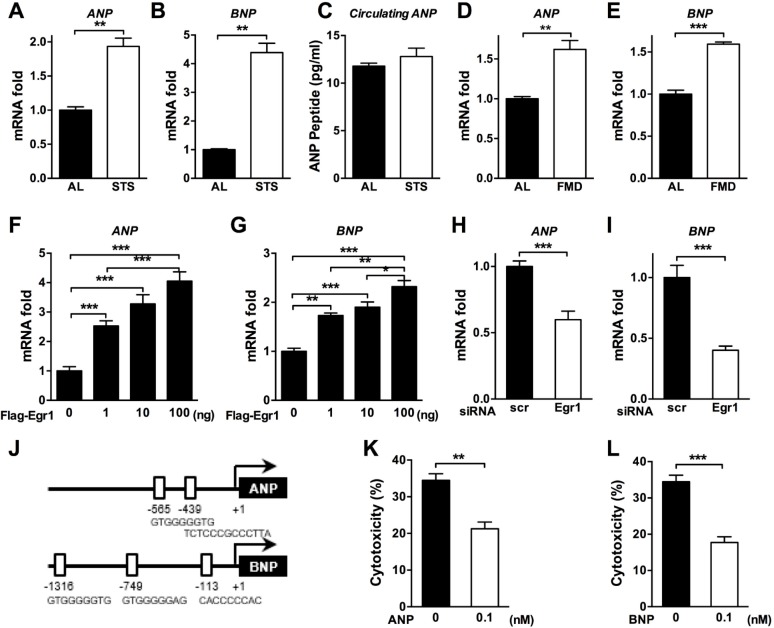
Fasting and a fasting-mimicking diet (FMD) induce the expression of cardiac peptide hormone atrial natriuretic peptide (ANP) and B-type natriuretic peptide (BNP) via early growth response protein 1 (EGR1). **(A, B, D,** and **E)** Fasting-regulated ANP and BNP expression. The heart tissues from short-term starvation (STS)- or FMD-treated mice were collected, and the expression levels of ANP and BNP were measured by real-time PCR. **(C)** Levels of ANP in peripheral blood of ad lib–fed and STS mice were assessed by ELISA. **(F-I)** EGR1-dependent ANP and BNP regulation. Primary rat cardiomyocytes were transfected with (**F, G)** a Flag-*Egr1* expression vector or **(H, I)** short interfering RNA (siRNA) against *Egr1*, and the relative mRNA levels were analyzed by real-time PCR for the indicated genes. **(J)** The putative EGR1 binding sites in the promoters of mouse ANP and BNP genes. **(K, L)** ANP and/or BNP-enhanced cardioprotection against doxorubicin (DXR). Primary cardiomyocytes were pretreated with 0.1 nM **(K)** ANP or **(L)** BNP for 24 h and treated with DXR the next day. Cytotoxicity was assessed 24 h after DXR treatment. Technical replicates are reported (*n* = 3 to 6), and data are represented as mean ± SEM. The significance of the differences between experimental groups was determined by using one-way ANOVA (Tukey post-analysis test). Comparisons between groups were performed with Student’s *t* test. *p*-values < 0.05 were considered significant (*p*-value < 0.05, 0.01, and 0.001 are indicated as *, *, and ***, respectively). Underlying data and method of statistical analysis are provided in S1 Data.

The atrial natriuretic peptide (ANP) and B-type natriuretic peptide (BNP) are members of the family of cardiac peptide hormones that are induced by cardiac stress and heart disease to protect cardiomyocytes [47]. To determine whether STS can regulate the expression levels of ANP and BNP genes, we analyzed heart tissue collected from mice undergoing ad lib feeding or a STS regimen. ANP (*p*-value < 0.001) and BNP (*p*-value < 0.001) expression was strongly induced in the heart of mice undergoing STS (Fig 3A and 3B**)**. Circulating ANP levels were not significantly altered, suggesting that local changes in the heart environment (autocrine and/or paracrine) rather than systemic changes (endocrine) could induce cellular protection (Fig 3C). As previously reported, a low-protein and low-carbohydrate FMD can be as effective as fasting in inducing DSR and in extending life span while maximizing nourishment and minimizing the risk of major adverse effects of complete food restriction [22, 24]. We tested the effect of this FMD on the in vivo regulation of ANP and BNP. Three cycles of the FMD regimen significantly induced the expression of ANP in the heart to the same extent observed in mice undergoing STS (*p*-value < 0.001) (Fig 3D). BNP was also induced by the FMD, although not at the same levels observed in animals undergoing complete fasting (*p*-value < 0.0001) (Fig 3E). These results indicate that both fasting and a fasting-mimicking diet positively control the expression of the cardioprotective ANP and BNP peptides in heart tissue.

We next tested whether the regulation of the cardiac peptides was mediated by EGR1. The overexpression of *Egr1* in cardiomyocytes induced the mRNA levels of the ANP and BNP genes in a dose-dependent manner (*p*-value < 0.0001) (Fig 3F and 3G). The role of EGR1 in the regulation of ANP and/or BNP was tested by treating cardiomyocytes with *Egr1* siRNA. The siRNA-mediated depletion of *Egr1* markedly reduced ANP and/or BNP expression levels (*p*-value < 0.0001; Fig 3H and 3I). Importantly, sequence analysis of the ANP and BNP gene promoters revealed that both contain several putative *Egr1* binding domains (Fig 3J). Pretreatment of primary cardiomyocytes with either ANP or BNP (0.1 nM) reduced DXR-induced cytotoxicity (*p*-value < 0.001 for ANP and *p*-value < 0.0001 for BNP) (Fig 3K and 3L), indicating that the STS-induced cardioprotection is mediated in part by the EGR1-dependent transcriptional regulation of ANP and BNP.

### Glucose restriction protects cardiomyocytes through AMPK/EGR1 activation

**Fig 4:**
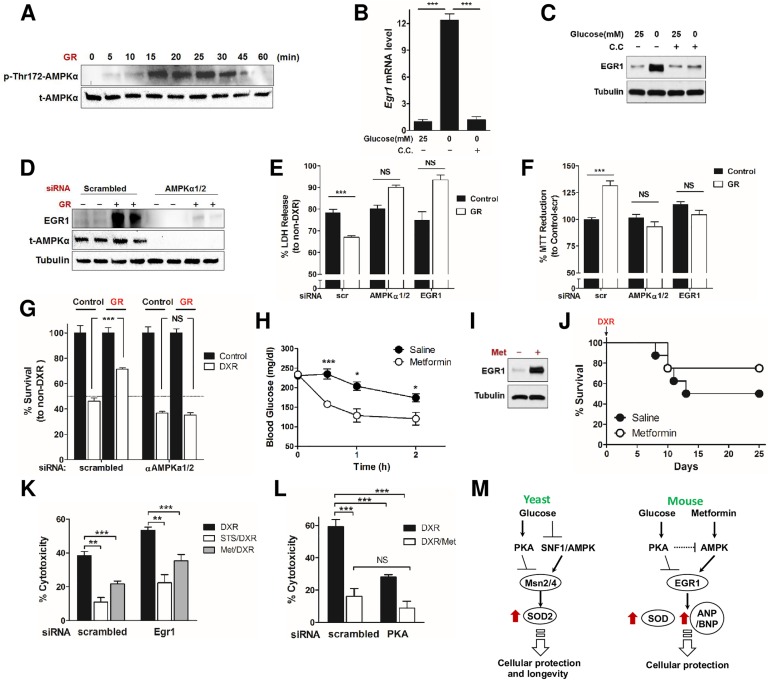
Early growth response protein 1 (EGR1) mediates metformin (Met)-induced stress resistance. **(A)** Time-dependent, glucose restriction (GR)-induced AMP-activated protein kinase (AMPK) activation was determined by the level of phosphorylation at Thr172 of AMPKα. **(B, C)** Primary rat cardiomyocytes were pretreated with or without compound C (20 μM) and then incubated with GR or normal medium. Whole-cell extracts were prepared and analyzed by **(B)** quantitative reverse transcription polymerase chain reaction (qRT-PCR) and **(C)** western blotting. **(D)** H9c2 cardiomyocytes transfected with siRNA against *AMPKα1/2* (or a nonspecific control) were treated with GR, and induction of EGR1 was determined by western blotting. (**E, F**) H9c2 cardiomyocytes were treated with siRNA against *AMPKα1/2* or *Egr1* or a nonspecific control and treated with doxorubicin (DXR). Cytotoxicity was determined by **(E)** % LDH release compared to corresponding non-DXR treated groups and **(F)** % 3-(4,5-dimethylthiazol-2-yl)-2,5-diphenyltetrazolium bromide (MTT) reduction compared to the group treated with nonspecific control siRNA not treated with DXR (*n* = 8). **(G)** H9c2 cardiomyocytes were treated with siRNA against *AMPKα1/2* or a nonspecific control and treated with and without GR and DXR. % survival was determined by trypan blue exclusion and normalized to each non-DXR treated group (*n* = 5–6). **(H)** Blood glucose levels from Met- and saline-injected mice were monitored for 2 h. **(I)** Primary rat cardiomyocytes were treated with Met (20 mM), and the expression level of EGR1 was analyzed by immunoblot. **(J)** Met-induced stress resistance. C57BL6 mice were injected daily with Met (50 mg/kg, intraperitoneal [IP]) for 2 wk prior to DXR administration (24 mg/kg). Survival was monitored for 25 d (*n* = 11; not statistically significant). **(K)** Cardiomyocytes transfected with siRNA against *PKA* were treated with Met (20 mM) and 24 h later were treated with DXR (1 μM). Cytotoxicity was measured 24 h after DXR treatment by LDH release to assess cell viability. **(L)** Cardiomyocytes transfected with siRNA against *PKA* (or a nonspecific control), were treated with Met (20 mM) and 24 h later were treated with DXR (1 μM). Cytotoxicity was measured 24 h after DXR treatment to assess cell viability. **(M)** A model for the starvation and/or Met-mediated AMPK/PKA-EGR1 (Msn2/4) pathways in yeast and mice. Both starvation (glucose restriction) and Met (Met in mice only) activate AMPK and inhibit protein kinase A (PKA), converging on *Egr1* induction (Msn2/4 in yeast). In agreement with other studies [36–38], PKA may negatively regulate AMPK. The consequent induction of *Egr1* or Msn2/4 triggers the expression of antioxidants, stress response genes (in both yeast and mice), and cardiac peptide hormones ANP/BNP, eventually enhancing cardioprotection (in mice). Technical replicates for the in vitro experiments are reported in B, E–G, K, and L (*n* = 3 to 4), and data are represented as mean ± SEM. The significance of the differences between experimental groups was determined by using one-way ANOVA (Tukey post-analysis test). Comparisons between groups were performed with Student’s *t* test. *p*-value < 0.05 were considered significant (*p*-value < 0.05, 0.01, and 0.001 are indicated as *, *, and ***, respectively). Underlying data and method of statistical analysis are provided in S1 Data.

AMPK is a master cellular energy sensor in most eukaryotic cells and, when activated by starvation, increases cellular protection [48–50] and induces *Egr1* expression [9, 34, 35]. Others have also shown that PKA can negatively regulate AMPK [36–38]. Here, we show that GR rapidly activates AMPK—as indicated by measuring the level of phosphorylation at Thr172—in rat cardiomyocytes (Fig 4A). To test whether EGR1 activation in response to GR is also mediated by AMPK, we treated cardiomyocytes with the AMPK-specific inhibitor compound C (CC) under normal and GR conditions. EGR1 induction by GR was abolished by CC treatment at the transcript (Fig 4B) and protein level (Fig 4C). Because CC is not entirely AMPK-specific [51], we also tested the effect of AMPK and *Egr1* inhibition using siRNA (S4 Fig). Knockdown of AMPKα1/2 in rat cardiomyocytes significantly inhibited GR-induced EGR1 expression (Fig 4D). Further, siRNA-mediated knockdown of AMPKα1/2 and *Egr1* reversed GR-mediated cardiomyocyte protection against DXR, determined by LDH release (Fig 4E) and 3-(4,5-dimethylthiazol-2-yl)-2,5-diphenyltetrazolium bromide (MTT) reduction (Fig 4F). The requirement for AMPKα1/2 for the GR-induced protection from DXR in cardiomyocytes was confirmed by trypan blue exclusion (Fig 4G). Conversely, the activation of AMPK by treatment with the pro-longevity diabetes drug metformin (Met) (50 mg/kg/day for 2 wk, intraperitoneal [IP]) significantly reduced blood glucose levels (Fig 4H and S2C Fig), induced *Egr1* expression in primary rat cardiomyocytes (Fig 4I and S2B Fig), and caused a nonsignificant trend for the protection of mice against DXR toxicity (Fig 4J). Such STS and/or Met-dependent protection against DXR was, in part, reversed by *Egr1* knockdown in rat cardiomyocytes (Fig 4K). Met and its analogue phenformin (Phen) both increased *Egr1* transcription in rat cardiomyocytes (S2B and S2D Fig). Met treatment also significantly increased *Egr1* activity in MEFs (S2F Fig). In addition, Met treatment increased the expression of EGR1 target genes catalase and Mn/CuZnSOD in mouse hearts (in vivo) and MnSOD and forkhead box protein O (FOXO)3a in MEFs (in vitro) (*p*-value < 0.0001; S2G–S2J Fig). We also found that *Egr1* is highly expressed at both the RNA and protein level in the heart of FMD-treated animals (*p*-value < 0.0001) (S3A and S3B Fig). FMD regimens also increased the expression of antioxidant enzymes, including catalase and Mn/CuZnSOD and FOXO1 and FOXO3 (*p*-value < 0.0001), which are key regulators of stress resistance and adaptive metabolic responses (S3C–S3G Fig) [52]. Because Met and nutrient restriction can induce AMPK activity but also to inhibit PKA activity [23, 53], we examined the combined effect of AMPK activation and PKA inhibition on DXR-induced cardiotoxicity. PKA knockdown alone protected cardiomyocytes from DXR toxicity but did not add to the protection provided by Met (Fig 4L), in accord with previous studies suggesting AMPK acts downstream of and is negatively regulated by PKA [36–38]. Further investigations will be necessary to determine if the cardioprotective effects of metformin against DXR in vivo are mediated by reduced blood glucose levels, its direct action on the heart, or a combination of both.

These findings suggest that GR-induced stress-resistance is mediated by a conserved pathway that involves PKA, AMPK, and transcription factors Msn2/4 (yeast) and *Egr1* (mice) (Fig 4M).

## Discussion

DXR is one of the most effective agents for the treatment of human breast, lung, thyroid, and ovarian carcinomas, but its therapeutic applications have been constrained by its dose-limiting side effects and, particularly, its cardiac toxicity [50, 37, 59]. Here, we provide evidence for the existence of a dexamethasone and glucose–PKA-dependent cellular sensitization conserved from yeast to mammalian cells, which is mediated in part by down-regulation of zinc finger transcription factor *Egr1* and its downstream effectors, including cardioprotective peptides ANP and BNP. This study indicates that dexamethasone and chemotherapy, which are widely administered in combination, enhance toxicity in mice, raising the possibility that patients receiving both could also be at risk.

The identification of the key players in STS-dependent cellular protection has been particularly challenging given the effect of fasting on a multitude of molecular and physiological changes, including the reduction of IGF-I and circulating glucose levels and the down-regulation of many signal transduction proteins, including mTOR and S6 kinase [28, 39]. Moreover, glucose homeostasis is regulated by a complex array of signals and cellular states, making it difficult to extrapolate the role of single components in cellular protection. Dexa and Rapa are given to cancer patients to reduce certain side effects of chemotherapy, enhance the killing of cancer cells, or both [54, 55]. However, our results indicate that the hyperglycemia induced by Dexa and Rapa is, at least in part, responsible for the increased mortality following high-dose DXR. These results are in agreement with the finding that high glucose levels in combination with chemotherapy in patients is associated with an increased risk of developing complicated infections and with a significant increase in overall mortality when compared with patients with normal blood glucose [56]. This is particularly concerning because Dexa is often used to overcome only minor side effects of chemotherapy such as nausea. Notably, a simple intervention such as STS, as well as insulin, prevented the hyperglycemia induced by Dexa or Rapa and reversed the sensitization effects in mice.

At the cellular level, GR and Met not only activate AMPK but also inhibit PKA, leading to EGR1-mediated protection of cardiomyocytes, possibly through the expression of antioxidant and stress resistance peptides. Met has been shown to preserve cardiac function through AMPK activation [57], and in fact, AMPK and PKA appear to have opposite effects on cardioprotection. Further investigations would be necessary to determine if Met is cardioprotective in vivo and if its effects against DXR are mediated by reduced blood glucose levels or direct actions on the heart or, possibly, a combination of both. MnSOD has been shown to have *Egr1* binding sites in its 5ʹ promoter region [58], in agreement with our finding here that EGR1 regulates MnSOD in response to GR, analogously to the effect of the ortholog transcription factors Msn2/4 in the regulation of MnSOD in yeast [59]. Notably, this study provides the first evidence for a pro-aging and/or cellular sensitization glucose–PKA pathway that inhibits zinc finger stress resistance transcription factors and downstream protective enzymes, including superoxide dismutase, conserved from yeast to mammals [23].

Interestingly, the conserved anti-aging forkhead stress resistance transcription factor FOXO, which has been shown to bind the MnSOD promoter and to regulate its expression [60], is not involved in the regulation of MnSOD during GR conditions (S5A–S5F Fig). Thus, the expression of MnSOD is transcriptionally up-regulated in response to GR and PKA inhibition in an EGR1-dependent but FOXO-independent manner.

Oxidative stress and the production of reactive oxygen species (ROS) during chemotherapy treatment can damage normal cells and are implicated in the toxicity of DXR [61]. Measurements of basal mitochondrial oxygen consumption rate (OCR) (as a measure of mitochondrial respiration), extracellular acidification rate (ECAR) (as a measure of glycolysis), and cellular ATP levels in *Egr1*^*+/+*^ and *Egr1*^-/-^ MEFs revealed that EGR1 is preferentially involved in the regulation of mitochondrial respiration without affecting ATP production, possibly suggesting a higher proton conductance across the mitochondrial inner membrane for *Egr1*^*+/+*^ cells (S5G–S5I Fig). Notably, in addition to activating AMPK, Met is known to inhibit respiration by targeting mitochondrial complex I [53]. These results indicate that EGR1 may contribute to improving mitochondrial efficiency, which may lower intracellular levels of ROS and protect against oxidative stress [62], although further studies are necessary to confirm this hypothesis.

ANP and B-type natriuretic peptide (BNP) are hormones produced in the heart that can regulate the intravascular blood volume. A rise of circulating ANP and BNP has been shown to compensate for heart failure because of their natriuretic, diuretic, and vasodilating actions. It has also been shown that natriuretic peptides have beneficial effects as therapeutic drugs in heart failure and acute myocardial infarction [63]. ANP and BNP can also act on the heart in an autocrine and/or paracrine fashion, as the heart expresses corresponding receptors [64, 65]. Local natriuretic peptides can also prevent cardiac hypertrophy [66, 67], which is a known consequence of DXR cardiotoxicity [68, 69]. Although fasting did not alter the circulating levels of ANP, it induced the expression of ANP and BNP in the heart. In cardiomyocytes, the expression of ANP and BNP was EGR1-mediated. Further animal studies are needed to confirm the role of EGR-1 and of ANP and/or BNP expression in mediating the cardioprotective effects of fasting against DXR.

Fasting induces a wide range of physiological responses, including nutrient and growth factor alterations [70]. Unlike yeast, mammalian systems utilize hormones, such as insulin, to regulate cellular functions in response to nutrient availability, raising the possibility that some of the effects associated with high glucose levels identified here could be caused by high levels of insulin and not hyperglycemia. However, both conditions in which glucose was low but insulin was either high (injection) or low (fasting) were protective, suggesting that it is the hypoglycemia and not the effects on insulin level that mediate the resistance to chemotherapy treatment. However, factors other than high glucose are likely to also contribute to the increased toxicity of chemotherapy observed after rapamycin or dexamethasone treatment. Further studies to carefully test further the effects of insulin, IGF-1, and other factors on stress resistance are necessary. In fact, our previous results indicate that reduction in IGF-1 levels are also important for protections against chemotherapy toxicity [28, 39].

In summary, our data show that the use of Dexa and Rapa, drugs widely used in cancer treatment which promote hyperglycemia, are associated with sensitization of mice and cardiomyocytes to the cytotoxic effects of DXR via the PKA–AMPK–EGR1 pathway. Together with human studies proposing that hyperglycemia may shorten the duration of remission for certain cancers, this study indicates that, unless the effects shown here are ruled out in humans, the clinical use of Dexa and other drugs promoting hyperglycemia in combination with chemotherapy drugs should be discontinued, particularly when it is only administered to prevent relatively minor side effects.

## Materials and methods

### Ethics statement

All of the experiments were approved by University of Southern California’s Institutional Animal Care and Use Committee (IACUC) before the experiments were started. All animals were kept in a positively pressurized, HEPA air-filtered animal room with 12-h light/dark cycles. Animals were anesthetized with 2% isoflurane during procedures.

### Starvation, fasting, and FMD treatment

For STS in vivo, mice were fasted for 24 to 72 h by complete deprivation of food but with free access to water. In order to avoid dehydration, the mice were fed a low-calorie hydrogel that encouraged water consumption, and body weight was measured immediately before, during, and after fasting. For a cell culture model, STS was performed by glucose and serum restriction. The culture media were supplemented with 0.5 g/L or 2.0 g/L glucose to match blood glucose levels in starved and normally fed mice, respectively [71]. FBS was supplemented at 1% for starvation conditions as compared to the normal 10%. In FMD conditions, mice were provided with less than 50% of the normal calorie intake for 4 d bimonthly, followed by ~60%–100% of the normal caloric intake.

### Animal studies

C57BL6 female mice were purchased from Charles River Laboratories International, Inc. or The Jackson Laboratory. Mice were maintained in a 12-h light/dark cycle at constant temperature and humidity. For stress resistance experiments, 12-wk-old female C57BL/6 mice were divided in the following experimental groups; ad lib (ad libitum feeding), STS, DXR, STS + DXR, Rapa, and STS + Rapa. It also included the following groups: Dexa, STS + Dexa, Insulin (Ins), Ins + Rapa, and Ins + Dexa.

Doxorubicin HCL was injected intravenously (IV). For the induction of heart toxicity, the animals were injected every 2 wk for a total of four injections with 8 mg/kg/mouse of DXR. For the stress resistance experiment, the mice were injected once with 24 mg/kg DXR. Rapa (1.5 mg/kg), Dexa (1 mg/kg), or Met (50 mg/kg) was administrated daily by intraperitoneal (IP) injection. Human normal insulin was administrated by IP injection twice a day for 2 consecutive days for a total of five injections.

The administration of Rapa and Dexa was performed for a period of 14 d after DXR administration. Insulin was administrated twice a day for a total of 48 h preceding the administration of DXR. Mice in the STS + DXR group were fed a very low-calorie and no-protein FMD for 48 h prior to the injection of DXR. Following DXR injection, the survival was recorded daily.

In order to induce hyperglycemia, glucose (2g/kg) was administrated intraperitoneally during the 24 h prior to DXR injection (Fig 1L and 1M) for a total of six injections. The last glucose injection was performed 1 h prior to DXR injection. Control animals were injected with 150 μl of sterile saline.

### Echocardiography

Two-dimensional echocardiography of the left ventricle (LV) on mice was performed using Vevo 770 Ultrasound system (Visualsonics). Mice were anesthetized with isoflurane and placed on a heating table in a supine position with four electrocardiography leads. Warmed ultrasound transmission gel (Parker Laboratories, cat. no. 01–20) was applied to the thorax surface to optimize the visibility of the chambers. The short-axis view of the LV was recorded to evaluate LV structure and diastolic function.

### Histopathological studies

Animals were anesthetized with 2% isoflurane, and the heart was collected and perfused with cold PBS. The LVs of heart tissues were extracted from mice and fixed in 4% neutral buffered formaldehyde. Sections of 5 um thickness were stained with hematoxylin (Sigma, cat. no. MHS16) for histological examination. In a blinded fashion, histomorphological evaluation of all the heart sections of mice was performed by a pathologist who was unaware of the groups.

### Cell culture

Cardiomyocytes were isolated from 1–3-d-old neonatal Sprague Dawley rats, which were purchased from Jackson. Tissue dissection was performed using Pierce Primary Cardiomyocyte Isolation Kit (Thermo Fisher Scientific, cat. no. 88281). H9c2 rat fetal cardiomyoblasts (ATCC, cat. no. CRL-1446) were grown in DMEM culture medium with high glucose (4.5 g/L; glucose concentration equivalent to ~25 mM), which was supplemented with 10% FBS. For glucose restriction studies, the cells were treated with 0.5 g/L glucose and pyruvate-free DMEM medium (Thermo Fisher Scientific, cat. no. A1443001), which was supplemented with 1% FBS for the indicated time.

### Transient transfection

Primary cardiomyocytes and H9C2 cells were transiently transfected in serum-free medium using the X-tremeGENE HP DNA Transfection Reagent (Roche Applied Science, cat. no. 06 366 236 001) with expression plasmid Flag-Egr1 [54].

For RNA interference, cells were transfected with Egr1 siRNA (SMARTpool, M-100247-01), PKA siRNA (SMARTpool, M-093299-02), and nontargeting control siRNA (SMARTpool, D-001210-01) obtained from Dharmacon and AMPKα1/2 siRNA (Santa Cruz, cat. no. sc-45312). Transfections of siRNA were performed using Lipofectamine RNAiMAX (Thermo Fisher Scientific, cat. no. 13778100) according to the manufacturer’s instructions.

### Luciferase reporter assay

Wild-type and DBE (DAF-16 family protein-binding element)-mt MnSOD-luc constructs [60] were gifted from Boudewijn M.T. Burgering at University Medical Center Utrecht, Utrecht, The Netherlands. FHRE-luc (#1789) [72] was purchased from Addgene.

### SEAP activity assay

Wild-type and Egr1-null MEFs were transfected with a reporter plasmid containing two repeats of EGR1-binding consensus sequence GCG GGG GCG driving the expression of SEAP. After 24 h, cells were placed in glucose restriction media or treated with the PKA inhibitor H-89 (Sigma, cat. no. B1427) for the indicated time. The media was collected and tested for SEAP activity using Great EscAPe SEAP Chemiluminescent Kit (Clontech, cat. no. 631736).

### Blood glucose measurements

Briefly, the tip of the tail was cut and 5 μl of blood were drawn directly in the glucose strip for the measurement (Precision Xtra, Abbott Diabetes Care Inc.).

### Bioenergetic measurements

Cells were seeded onto an XF96 cell culture microplate (Seahorse Bioscience) at 2˗3 × 10^4^ cells/well. Metabolic rates were measured using an XF96 Extracellular Flux Analyzer (Seahorse Bioscience). XF Cell Mito Stress and XF Glycolysis Stress Test kits were used to measure the key parameters of mitochondrial functions and cellular glycolysis, respectively, according to the manufacturer’s instructions (Seahorse Bioscience).

### LDH release assay

Primary cardiomyocytes and H9C2 cells were seeded in 96-well culture plates (1 × 106 cells/well) and transfected with siRNA of Egr1, AMPKα 1/2, and nontargeting control or treated with ANP or BNP peptide. For siRNA-transfected cells, at 24 h posttransfection with siRNA, the cells were placed in glucose restriction media, and at 48 h posttransfection, the cells were treated with 1 uM of doxorubicin (Sigma, cat. no. D1515) for 24 h. For the pretreatment of peptides, ANP or BNP peptide was treated for 24 h. To quantify the release of LDH from the cells, the CytoTox 96 Non-Radioactive Cytotoxicity Assay (Promega, cat. no. G1780) was used according to the manufacturer’s instruction. The percent release was normalized to detergent lysis (2% Triton X-100) treated cells for peptide pretreatment and non-DXR treated cells.

### MTT assay

H9C2 cells were seeded with culture medium in 96-well culture plates and incubated at 37°C for 24 h before siRNA transfection. As described above, at 24 h after treatments with doxorubicin, the siRNA-transfected cells were incubated with culture medium containing 0.4 mg/ml MTT (3-(4,5-dimethylthiazol-2-yl)- 2,5-diphenyltetrazolium bromide (Sigma-Aldrich, cat. no. M5655) at 37°C for 4 h. The reduced formazan crystals were dissolved in SDS and the absorbance was detected at 570 nm against 660 nm.

### Trypan blue exclusion assay

H9C2 were seeded in 12-well culture plates (0.1 × 10^6^ cells/well) and transfected with siRNA of AMPKα 1/2 or nontargeting control for 24 h. At 24 h posttransfection, glucose restriction media was replaced, and at 48 h posttransfection, doxorubicin was treated for 24 h as described above. Cells were trypsinized, and cell viability was determined using a trypan blue dye (0.4% trypan blue solution [Thermo Fisher Scientific, cat. no. 15250061]). The number of viable cells was subsequently counted under light microscope.

### PKA kinase activity

PKA kinase activity was assessed in vitro by colorimetric assay (Enzo Biochem, Inc. cat no. ADI-EKS-390A).

### Western blot analysis

Immunoblot analysis was performed as previously described [73]. Briefly, the cells were lysed in modified RIPA buffer containing 50 mM HEPES, 0.3% NP-40, 75 mM NaCl, 0.1% SDS, 1% sodium deoxycholate, and EDTA-free protease (Thermo Fisher Scientific, cat. no. 88266) and phosphatase inhibitors (Thermo Fisher Scientific, cat. no. 88667) on ice. 35 ug of proteins were resolved using 4%–20% Mini-PROTEAN TGX Precast Protein Gels (Bio-Rad, cat. no. 4561096) and transferred to PVDF membranes (Bio-Rad, cat. no. 1620177). The transferred blot was blocked with 5% BSA for 30 min in TBS containing 0.05% Tween-20 at room temperature for 30 min. Then the membranes were immunoblotted with primary antibody Egr1 (Santa Cruz, cat. no. sc-189; 1:500), phospho-AMPKα (Thr172) (Cell Signaling, cat. no. 2535; 1:1,000), AMPKα 1/2 (total) (Cell Signaling, cat. no. 2532; 1:1,000), AMPKα2 (total) (Cell Signaling, cat. no. 2757; 1:1,000), and β-tubulin (Cell Signaling, cat. no. 2146; 1:1,000) at 4°C overnight.

### RNA isolation and quantitative RT-PCR

Total RNA was isolated using TRIzol (Thermo Fisher Scientific, cat. no. 15596026). Then, RNA was reverse transcribed using M-MLV Reverse Transcriptase (Promega, cat. no. M170A) and then amplified with SYBR Select Master Mix (Thermo Fisher Scientific, cat. no. 447903) according to the manufacturer’s instructions. Quantitative RT-PCR was performed using QuantStudio 6 Flex Real-Time PCR System (Thermo Fisher Scientific, cat. no. 4485692) and the primers Egr1: 5ʹ-GACGAGTTATCCCAGCCAA-3ʹ (forward) and 5ʹ-GGCAGAGGAAGACGA TGAAG-3ʹ (reverse); Catalase: 5ʹ-CCTGACATGGTCTGGGACTT-3ʹ (forward) and 5ʹ-CAAGTTTTTGATGCCCTGGT-3ʹ (reverse); MnSOD: 5ʹ-CCAGTGCAGGACCTCATTTT-3ʹ (forward) and 5ʹ-AGACAGGCAAGGCTCTACCA-3ʹ (reverse); FOXO1: 5ʹ-TAACTGTGCCCCAGGACTCT-3ʹ (forward) and 5ʹ-AGCTGGGGTTCATCATTTTG-3ʹ (reverse); FOXO3: 5ʹ-GGGGAGTTTGGTCAATCAGA-3ʹ (forward) and 5ʹ-GCCTGAGAGAGAGTCCGAGA-3ʹ (reverse); and β-Actin: 5ʹ-(primer sequence). β-Actin was used as a reference gene for normalization of target genes.

### Drug administration

#### Rapamycin

Rapamycin (LC Laboratories) stock solution was prepared by dissolving the compound in DMSO to a final concentration of 50 mg/ml. Single aliquots of the stock solution were stored at –20°C. For in vivo use, rapamycin 1.5 mg/kg was administrated daily by IP injection. Before injection, rapamycin working solution was freshly made by dissolving the stock solution in 5% PEG, 5% TWEEN 80, and 4% ethanol in sterile saline. For in vitro use, rapamycin stock solution was serially diluted in DMEM cell culture medium (Dulbecco's Modified Eagle's Medium—Life Technologies) at a final concentration of 5 uM for ACN, HTLA-230, NXS2, and SH-SY5Y cells and to 10 uM for 4T1 and MEF cells.

#### Dexamethasone

Stock solution of dexamethasone (Sigma-Aldrich Co. LLC) was prepared by dissolving the compound in 100% ethanol to a final concentration of 25 mg/ml. The working solution was freshly prepared every day by dissolving the stock solution in saline and administrated intraperitoneally (1 mg/kg).

#### Metformin

Metformin was purchased from Sigma-Aldrich Co LCC. The powder was dissolved in saline to make a stock solution that was stored at –20°C. The working solution was freshly made every day, and each animal was injected intraperitoneally with metformin 50 mg/kg (roughly 100 μl per animal)

#### Insulin

Human normal insulin was administrated twice a day for two consecutive days for a total of five injections. Stock solution of insulin was diluted in sterile saline, and 1.5 U/kg per mouse were administrated by IP injection.

### Yeast Study

Wild-type and *Δmsn2/4* double mutant yeast stains (all derivatives of DBY746 *MATα leu2-3*, *112*, *his3*Δ, *trp1-289*, *ura3-52*, *GAL*^*+*^) were streaked out from frozen stock onto YPD plates and incubated at 30°C for 2 d. Next, 3–5 colonies were inoculated in 2 ml of liquid SDC and incubated overnight. 100 μl of the overnight culture was added to four 50-ml flasks containing 10 ml of fresh SDC with different glucose concentrations (i.e., 0.5%, 2%, 5%, or 10%) and incubated at 30°C for 4 d. Following the incubation period, 1 OD_600_ aliquot from each flask was treated with 200 μM menadione, 100 mM H_2_O_2_, or buffer only in 0.1 M KPO_4_ buffer and incubated at 30°C for 1 h. Posttreatment, a 10-fold serial dilution was spotted on YPD plates and incubated at 30°C for 4 d.

### Statistics

ImageJ software was used for the quantification of the signal and GraphPad Prism v5.0c was used for the graphic representation and statistical analysis of the data. The significance of the differences between experimental groups was determined by using Analysis Of Variance (ANOVA) analysis in GraphPad Prism v5.0c. Tukey test was used in the post-analysis, and the differences were considered significant if the *p-*value was ≤0.05. Comparisons between groups were done with Student’s *t* test using GraphPad Prism v.5.0c. All the statistical analyses were two-sided, and *p-*values ≤ 0.05 were considered significant.

## Supporting information

S1 Fig Experimental schematic representation for the induction of heart damage in vivo and for STS, dexamethasone/rapamycin treatments with doxorubicin. (**A**) C57BL/6 mice were divided into 4 groups and underwent *ad libitum* feeding (ad lib), STS (48 h fasting) and/or doxorubicin treatment (DXR, 8 mg/kg). DXR was administrated at the end of each STS cycle. (**B**) Mice were daily injected with dexamethasone or rapamycin for 2 weeks, before undergoing STS regimen (48h). Insulin group was treated with 1.5 U of insulin twice a day for 2 days before DXR (24 mg/kg) administration at the end of STS. Their survival was monitored for up to 35 days.
(TIF)

S2 Fig AMPK activation by GR, metformin, and its analog phenformin induce the expression of Egr1, MnSOD, and FoxO downstream genes. (**A**, **B**) H9c2 cardiomyocytes transfected with *Egr1*-promoter luciferase were treated with GR or metformin (20 mM). Luciferase activity was carried out 6 h post-treatment. (**C**) Body weight of mice undergoing control and metformin treatment was monitored to exclude body weight loss due to drug administration. Points and bars represent the mean ±s.e.m. (n = 10). (**D**) H9c2 cardiomyocytes were treated with phenformin (2 mM) for 1 h and *Egr1* mRNA levels were measured using qRT-PCR. (**E**) *Egr1*^*+/+*^ and *Egr1*^*-/-*^ MEFs and their response to glucose restriction (GR). (**F-J**) *Egr1*^*+/+*^ and *Egr1*^*-/-*^ MEFs were treated with Met following transient transfection with (**G**) EGR1-SEAP reporter (SEAP activity was measured and assessed over time by using a chemiluminescent assay), (**H-J**) MnSOD-luc, MnSOD-mt-luc, or FHRE-luc (luciferase activity was measured 18 h after treatment). P-value <0.05 were considered significant (p-value<0.05, 0.01 and 0.001 are indicated as *, *, and ***, respectively). Underlying data and method of statistical analysis are provided in S1 Data.
(TIF)

S3 Fig FMD induces the expression of Egr1 and antioxidant genes. (**A**-**G**) Heart tissues from *ad lib* (AL) and FMD-treated mice were collected and protein and mRNA levels for the indicated genes were assessed by (**A**) Western blotting and (**B-G**) qRT-PCR, respectively. P-value <0.05 were considered significant (p-value<0.05, 0.01 and 0.001 are indicated as *, *, and ***, respectively). Underlying data and method of statistical analysis are provided in S1 Data.
(TIF)

S4 Fig siRNA-mediated knock-down of (A) EGR1 and (B) AMPKα2 and AMPKα1/2 in H9c2 cells following glucose restriction.
(TIF)

S5 Fig Cellular respiration, MnSOD, and FOXO responsive genes are regulated in a PKA/EGR1-dependent manner. (**A**-**C**). *Egr1*^*+/+*^ and *Egr1*^*-/-*^ MEFs were transfected with (**A**) FHRE-luc, (**B**) MnSOD-mt-luc, or (**C**) MnSOD-luc, and treated with H89. Luciferase activity was assessed 18 h after treatment. (**D**-**F**) The regulation of MnSOD and FOXO responsive genes in Egr1-dependent manner. *Egr1*^*+/+*^ and *Egr1*^*-/-*^ MEFs were transfected with (**D**) FHRE-luc, (**E**) MnSOD-mt-luc, or (**F**) MnSOD-luc, and treated with GR medium. Luciferase activity was measured 18 h after treatment. (**G**-**I**) EGR1 regulates energy metabolism. (**G**) Basal levels of OCR to ECAR ratios, (**H**) ATP production, and (**I**) proton conductance were measured in *Egr1*^*+/+*^ and *Egr1*^*-/-*^ MEFs by the XF96 extracellular flux analyzer. P-value <0.05 were considered significant (p-value<0.05, 0.01 and 0.001 are indicated as *, *, and ***, respectively). Underlying data and method of statistical analysis are provided in S1 Data.
(TIF)

S1 Data Underlying data and method of statistical analysis. (XLSX)

## Abbreviations

AL: ad lib
AMPK: AMP-activated protein kinase
ANP: atrial natriuretic peptide
AU: arbitrary unit
BNP: B-type natriuretic peptide
CC: compound C
Dexa: dexamethasone
DSR: differential stress resistance
DXR: doxorubicin
ECAR: extracellular acidification rate
EGR1: early growth response protein 1
FBS: fetal bovine serum
FMD: fasting-mimicking diet
FOXO: forkhead box protein O
GR: glucose restriction
IP: intraperitoneal
IV: intravenous
LDH: lactate dehydrogenase
LV: left ventricle
MEF: mouse embryonic fibroblast
Met: metformin
mTOR: mammalian target of rapamycin
MTT: 3-(4,5-dimethylthiazol-2-yl)-2,5-diphenyltetrazolium bromide
OCR: oxygen consumption rate
Phen: phenformin
PKA: protein kinase A
PNET: primitive neuroectodermal tumor
qRT-PCR: quantitative reverse transcription polymerase chain reaction
Rapa: rapamycin
RCC: renal cell carcinoma
ROS: reactive oxygen species
SEAP: secreted alkaline phosphatase
SEGA: subependymal giant cell astrocytoma
siRNA: short interfering RNA
STS: short-term starvation
YPD: yeast extract peptone dextrose

## References

[1] Takemura G, Fujiwara H. Doxorubicin-induced cardiomyopathy from the cardiotoxic mechanisms to management. Progress in cardiovascular diseases. 2007 Mar-Apr;49(5):330–52. 10.1016/j.pcad.2006.10.002 17329180

[2] Tucker MA, Jones PH, Boice JD Jr., Robison LL, Stone BJ, Stovall M, et al Therapeutic radiation at a young age is linked to secondary thyroid cancer. The Late Effects Study Group. Cancer research. 1991 6 1;51(11):2885–8. 1851664

[3] Schneider U, Kaser-Hotz B. A simple dose-response relationship for modeling secondary cancer incidence after radiotherapy. Zeitschrift fur medizinische Physik. 2005;15(1):31–7. 15830782 10.1078/0939-3889-00242

[4] Bhatia S, Yasui Y, Robison LL, Birch JM, Bogue MK, Diller L, et al High risk of subsequent neoplasms continues with extended follow-up of childhood Hodgkin's disease: report from the Late Effects Study Group. Journal of clinical oncology: official journal of the American Society of Clinical Oncology. 2003 12 1;21(23):4386–94. 14645429 10.1200/JCO.2003.11.059

[5] Steinherz LJ, Steinherz PG, Tan CT, Heller G, Murphy ML. Cardiac toxicity 4 to 20 years after completing anthracycline therapy. Jama. 1991 9 25;266(12):1672–7. 1886191

[6] Zhang S, Liu X, Bawa-Khalfe T, Lu LS, Lyu YL, Liu LF, et al Identification of the molecular basis of doxorubicin-induced cardiotoxicity. Nature medicine. 2012 11;18(11):1639–42. 10.1038/nm.2919 23104132

[7] Herrstedt J, Aapro MS, Roila F, Kataja VV. ESMO Minimum Clinical Recommendations for prophylaxis of chemotherapy-induced nausea and vomiting (NV). Annals of oncology: official journal of the European Society for Medical Oncology/ESMO. 2005;16 Suppl 1:i77–9. 10.1093/annonc/mdi805 15888767

[8] Jordan K, Kasper C, Schmoll HJ. Chemotherapy-induced nausea and vomiting: current and new standards in the antiemetic prophylaxis and treatment. Eur J Cancer. 2005 1;41(2):199–205. 10.1016/j.ejca.2004.09.026 15661543

[9] Benboubker L, Dimopoulos MA, Dispenzieri A, Catalano J, Belch AR, Cavo M, et al Lenalidomide and dexamethasone in transplant-ineligible patients with myeloma. The New England journal of medicine. 2014 9 4;371(10):906–17. 10.1056/NEJMoa1402551 25184863

[10] Lendvai N, Hilden P, Devlin S, Landau H, Hassoun H, Lesokhin AM, et al A phase 2 single-center study of carfilzomib 56 mg/m2 with or without low-dose dexamethasone in relapsed multiple myeloma. Blood. 2014 8 7;124(6):899–906. 10.1182/blood-2014-02-556308 24963043 PMC4624439

[11] Gisselbrecht C. Should We Replace Dexamethasone, Cytarabine, and Cisplatin for Relapsed Lymphoma? Journal of clinical oncology: official journal of the American Society of Clinical Oncology. 2014 9 29;32(31):3472–3. 25267756 10.1200/JCO.2014.56.6802

[12] Samuels AL, Beesley AH, Yadav BD, Papa RA, Sutton R, Anderson D, et al A pre-clinical model of resistance to induction therapy in pediatric acute lymphoblastic leukemia. Blood cancer journal. 2014;4:e232 10.1038/bcj.2014.52 25083816 PMC4219466

[13] Lukins MB, Manninen PH. Hyperglycemia in patients administered dexamethasone for craniotomy. Anesthesia and analgesia. 2005 4;100(4):1129–33. 10.1213/01.ANE.0000146943.45445.55 15781533

[14] Bostrom B, Uppal P, Chu J, Messinger Y, Gandrud L, McEvoy R. Safety and efficacy of metformin for therapy-induced hyperglycemia in children with acute lymphoblastic leukemia. Journal of pediatric hematology/oncology. 2013 10;35(7):504–8. 10.1097/MPH.0b013e31829cdeba 23823111

[15] Leib SL, Heimgartner C, Bifrare YD, Loeffler JM, Taauber MG. Dexamethasone aggravates hippocampal apoptosis and learning deficiency in pneumococcal meningitis in infant rats. Pediatric research. 2003 9;54(3):353–7. 10.1203/01.PDR.0000079185.67878.72 12788989

[16] Poungvarin N, Bhoopat W, Viriyavejakul A, Rodprasert P, Buranasiri P, Sukondhabhant S, et al Effects of dexamethasone in primary supratentorial intracerebral hemorrhage. The New England journal of medicine. 1987 5 14;316(20):1229–33. 10.1056/NEJM198705143162001 3574383

[17] Seto B. Rapamycin and mTOR: a serendipitous discovery and implications for breast cancer. Clinical and translational medicine. 2012;1(1):29 10.1186/2001-1326-1-29 23369283 PMC3561035

[18] Motzer RJ, Escudier B, Oudard S, Hutson TE, Porta C, Bracarda S, et al Efficacy of everolimus in advanced renal cell carcinoma: a double-blind, randomised, placebo-controlled phase III trial. Lancet. 2008 8 9;372(9637):449–56. 10.1016/S0140-6736(08)61039-9 18653228

[19] Hudes G, Carducci M, Tomczak P, Dutcher J, Figlin R, Kapoor A, et al Temsirolimus, interferon alfa, or both for advanced renal-cell carcinoma. The New England journal of medicine. 2007 5 31;356(22):2271–81. 10.1056/NEJMoa066838 17538086

[20] Busaidy NL, Farooki A, Dowlati A, Perentesis JP, Dancey JE, Doyle LA, et al Management of metabolic effects associated with anticancer agents targeting the PI3K-Akt-mTOR pathway. Journal of clinical oncology: official journal of the American Society of Clinical Oncology. 2012 8 10;30(23):2919–28. 22778315 10.1200/JCO.2011.39.7356 PMC3410405

[21] Houde VP, Brule S, Festuccia WT, Blanchard PG, Bellmann K, Deshaies Y, et al Chronic rapamycin treatment causes glucose intolerance and hyperlipidemia by upregulating hepatic gluconeogenesis and impairing lipid deposition in adipose tissue. Diabetes. 2010 6;59(6):1338–48. 10.2337/db09-1324 20299475 PMC2874694

[22] Longo VD, Mattson MP. Fasting: Molecular Mechanisms and Clinical Applications. Cell metabolism. 2014 2 4;19(2):181–92. 10.1016/j.cmet.2013.12.008 24440038 PMC3946160

[23] Fontana L, Partridge L, Longo VD. Extending healthy life span—from yeast to humans. Science. 2010 4 16;328(5976):321–6. 10.1126/science.1172539 20395504 PMC3607354

[24] Brandhorst S, Choi IY, Wei M, Cheng CW, Sedrakyan S, Navarrete G, et al A Periodic Diet that Mimics Fasting Promotes Multi-System Regeneration, Enhanced Cognitive Performance, and Healthspan. Cell metabolism. 2015 7 7;22(1):86–99. 10.1016/j.cmet.2015.05.012 26094889 PMC4509734

[25] Crouthamel MC, Kahana JA, Korenchuk S, Zhang SY, Sundaresan G, Eberwein DJ, et al Mechanism and management of AKT inhibitor-induced hyperglycemia. Clinical cancer research: an official journal of the American Association for Cancer Research. 2009 1 1;15(1):217–25. 19118049 10.1158/1078-0432.CCR-08-1253

[26] Di Biase S, Lee C, Brandhorst S, Manes B, Buono R, Cheng CW, et al Fasting-Mimicking Diet Reduces HO-1 to Promote T Cell-Mediated Tumor Cytotoxicity. Cancer cell. 2016 7 11;30(1):136–46. 10.1016/j.ccell.2016.06.005 27411588 PMC5388544

[27] Josefsen K, Sorensen LR, Buschard K, Birkenbach M. Glucose induces early growth response gene (Egr-1) expression in pancreatic beta cells. Diabetologia. 1999 2;42(2):195–203. 10.1007/s001250051139 10064100

[28] Raffaghello L, Lee C, Safdie FM, Wei M, Madia F, Bianchi G, et al Starvation-dependent differential stress resistance protects normal but not cancer cells against high-dose chemotherapy. Proceedings of the National Academy of Sciences of the United States of America. 2008 6 17;105(24):8215–20. 10.1073/pnas.0708100105 18378900 PMC2448817

[29] Wei M, Fabrizio P, Hu J, Ge H, Cheng C, Li L, et al Life span extension by calorie restriction depends on Rim15 and transcription factors downstream of Ras/PKA, Tor, and Sch9. PLoS Genet. 2008 1;4(1):e13 10.1371/journal.pgen.0040013 18225956 PMC2213705

[30] Estruch F, Carlson M. Two homologous zinc finger genes identified by multicopy suppression in a SNF1 protein kinase mutant of Saccharomyces cerevisiae. Molecular and cellular biology. 1993 7;13(7):3872–81. 8321194 10.1128/mcb.13.7.3872-3881.1993 PMC359918

[31] Yu J, Zhang SS, Saito K, Williams S, Arimura Y, Ma Y, et al PTEN regulation by Akt-EGR1-ARF-PTEN axis. The EMBO journal. 2009 1 7;28(1):21–33. 10.1038/emboj.2008.238 19057511 PMC2633077

[32] Sukhatme VP, Cao XM, Chang LC, Tsai-Morris CH, Stamenkovich D, Ferreira PC, et al A zinc finger-encoding gene coregulated with c-fos during growth and differentiation, and after cellular depolarization. Cell. 1988 4 8;53(1):37–43. 3127059 10.1016/0092-8674(88)90485-0

[33] Baron V, Adamson ED, Calogero A, Ragona G, Mercola D. The transcription factor Egr1 is a direct regulator of multiple tumor suppressors including TGFbeta1, PTEN, p53, and fibronectin. Cancer gene therapy. 2006 2;13(2):115–24. 10.1038/sj.cgt.7700896 16138117 PMC2455793

[34] Berasi SP, Huard C, Li D, Shih HH, Sun Y, Zhong W, et al Inhibition of gluconeogenesis through transcriptional activation of EGR1 and DUSP4 by AMP-activated kinase. The Journal of biological chemistry. 2006 9 15;281(37):27167–77. 10.1074/jbc.M602416200 16849326

[35] Andrade J, Quinn J, Becker RZ, Shupnik MA. AMP-activated protein kinase is a key intermediary in GnRH-stimulated LHbeta gene transcription. Mol Endocrinol. 2013 5;27(5):828–39. 10.1210/me.2012-1323 23518923 PMC3634116

[36] Hurley RL, Barre LK, Wood SD, Anderson KA, Kemp BE, Means AR, et al Regulation of AMP-activated protein kinase by multisite phosphorylation in response to agents that elevate cellular cAMP. The Journal of biological chemistry. 2006 12 1;281(48):36662–72. 10.1074/jbc.M606676200 17023420

[37] Djouder N, Tuerk RD, Suter M, Salvioni P, Thali RF, Scholz R, et al PKA phosphorylates and inactivates AMPKalpha to promote efficient lipolysis. The EMBO journal. 2010 1 20;29(2):469–81. 10.1038/emboj.2009.339 19942859 PMC2824464

[38] Iwata T, Taniguchi H, Kuwajima M, Taniguchi T, Okuda Y, Sukeno A, et al The action of D-dopachrome tautomerase as an adipokine in adipocyte lipid metabolism. PLoS ONE. 2012;7(3):e33402 10.1371/journal.pone.0033402 22428043 PMC3299789

[39] Lee C, Safdie FM, Raffaghello L, Wei M, Madia F, Parrella E, et al Reduced levels of IGF-I mediate differential protection of normal and cancer cells in response to fasting and improve chemotherapeutic index. Cancer research. 2010 2 15;70(4):1564–72. 10.1158/0008-5472.CAN-09-3228 20145127 PMC2836202

[40] Lamming DW, Ye L, Katajisto P, Goncalves MD, Saitoh M, Stevens DM, et al Rapamycin-induced insulin resistance is mediated by mTORC2 loss and uncoupled from longevity. Science. 2012 3 30;335(6076):1638–43. 10.1126/science.1215135 22461615 PMC3324089

[41] Houstis N, Rosen ED, Lander ES. Reactive oxygen species have a causal role in multiple forms of insulin resistance. Nature. 2006 4 13;440(7086):944–8. 10.1038/nature04634 16612386

[42] Waldron NH, Jones CA, Gan TJ, Allen TK, Habib AS. Impact of perioperative dexamethasone on postoperative analgesia and side-effects: systematic review and meta-analysis. British journal of anaesthesia. 2013 2;110(2):191–200. 10.1093/bja/aes431 23220857 PMC3544008

[43] Feldman-Billard S, Du Pasquier-Fediaevsky L, Heron E. Hyperglycemia after repeated periocular dexamethasone injections in patients with diabetes. Ophthalmology. 2006 10;113(10):1720–3. 10.1016/j.ophtha.2006.05.023 17011953

[44] Andrikopoulos S, Blair AR, Deluca N, Fam BC, Proietto J. Evaluating the glucose tolerance test in mice. American journal of physiology Endocrinology and metabolism. 2008 12;295(6):E1323–32. 10.1152/ajpendo.90617.2008 18812462

[45] Cheng CW, Adams GB, Perin L, Wei M, Zhou X, Lam BS, et al Prolonged fasting reduces IGF-1/PKA to promote hematopoietic-stem-cell-based regeneration and reverse immunosuppression. Cell stem cell. 2014 6 5;14(6):810–23. 10.1016/j.stem.2014.04.014 24905167 PMC4102383

[46] Almeida MQ, Tsang KM, Cheadle C, Watkins T, Grivel JC, Nesterova M, et al Protein kinase A regulates caspase-1 via Ets-1 in bone stromal cell-derived lesions: a link between cyclic AMP and pro-inflammatory pathways in osteoblast progenitors. Human molecular genetics. 2011 1 1;20(1):165–75. 10.1093/hmg/ddq455 20940146 PMC3000682

[47] Burley DS, Hamid SA, Baxter GF. Cardioprotective actions of peptide hormones in myocardial ischemia. Heart failure reviews. 2007 12;12(3–4):279–91. 10.1007/s10741-007-9029-y 17516166

[48] Hardie DG, Ross FA, Hawley SA. AMPK: a nutrient and energy sensor that maintains energy homeostasis. Nat Rev Mol Cell Biol. 2012 4;13(4):251–62. 10.1038/nrm3311 22436748 PMC5726489

[49] Kroemer G, Marino G, Levine B. Autophagy and the integrated stress response. Mol Cell. 2010 10 22;40(2):280–93. 10.1016/j.molcel.2010.09.023 20965422 PMC3127250

[50] Shaw RJ, Kosmatka M, Bardeesy N, Hurley RL, Witters LA, DePinho RA, et al The tumor suppressor LKB1 kinase directly activates AMP-activated kinase and regulates apoptosis in response to energy stress. Proceedings of the National Academy of Sciences of the United States of America. 2004 3 9;101(10):3329–35. 10.1073/pnas.0308061100 14985505 PMC373461

[51] Bain J, Plater L, Elliott M, Shpiro N, Hastie CJ, McLauchlan H, et al The selectivity of protein kinase inhibitors: a further update. The Biochemical journal. 2007 12 15;408(3):297–315. 10.1042/BJ20070797 17850214 PMC2267365

[52] Calnan DR, Brunet A. The FoxO code. Oncogene. 2008 4 7;27(16):2276–88. 10.1038/onc.2008.21 18391970

[53] Pernicova I, Korbonits M. Metformin—mode of action and clinical implications for diabetes and cancer. Nature reviews Endocrinology. 2014 3;10(3):143–56. 10.1038/nrendo.2013.256 24393785

[54] Wang H, Li M, Rinehart JJ, Zhang R. Pretreatment with dexamethasone increases antitumor activity of carboplatin and gemcitabine in mice bearing human cancer xenografts: in vivo activity, pharmacokinetics, and clinical implications for cancer chemotherapy. Clinical cancer research: an official journal of the American Association for Cancer Research. 2004 3 1;10(5):1633–44. 15014014 10.1158/1078-0432.ccr-0829-3

[55] Mondesire WH, Jian W, Zhang H, Ensor J, Hung MC, Mills GB, et al Targeting mammalian target of rapamycin synergistically enhances chemotherapy-induced cytotoxicity in breast cancer cells. Clinical cancer research: an official journal of the American Association for Cancer Research. 2004 10 15;10(20):7031–42. 15501983 10.1158/1078-0432.CCR-04-0361

[56] Weiser MA, Cabanillas ME, Konopleva M, Thomas DA, Pierce SA, Escalante CP, et al Relation between the duration of remission and hyperglycemia during induction chemotherapy for acute lymphocytic leukemia with a hyperfractionated cyclophosphamide, vincristine, doxorubicin, and dexamethasone/methotrexate-cytarabine regimen. Cancer. 2004 3 15;100(6):1179–85. 10.1002/cncr.20071 15022284

[57] Xie Z, Lau K, Eby B, Lozano P, He C, Pennington B, et al Improvement of cardiac functions by chronic metformin treatment is associated with enhanced cardiac autophagy in diabetic OVE26 mice. Diabetes. 2011 6;60(6):1770–8. 10.2337/db10-0351 21562078 PMC3114402

[58] Porntadavity S, Xu Y, Kiningham K, Rangnekar VM, Prachayasittikul V, St Clair DK. TPA-activated transcription of the human MnSOD gene: role of transcription factors Sp-1 and Egr-1. DNA and cell biology. 2001 8;20(8):473–81. 10.1089/104454901316976109 11560779

[59] Sadeh A, Movshovich N, Volokh M, Gheber L, Aharoni A. Fine-tuning of the Msn2/4-mediated yeast stress responses as revealed by systematic deletion of Msn2/4 partners. Molecular biology of the cell. 2011 9;22(17):3127–38. 10.1091/mbc.E10-12-1007 21757539 PMC3164460

[60] Kops GJ, Dansen TB, Polderman PE, Saarloos I, Wirtz KW, Coffer PJ, et al Forkhead transcription factor FOXO3a protects quiescent cells from oxidative stress. Nature. 2002 9 19;419(6904):316–21. 10.1038/nature01036 12239572

[61] Conklin KA. Dietary antioxidants during cancer chemotherapy: impact on chemotherapeutic effectiveness and development of side effects. Nutrition and cancer. 2000;37(1):1–18. 10.1207/S15327914NC3701_1 10965514

[62] Divakaruni AS, Brand MD. The regulation and physiology of mitochondrial proton leak. Physiology (Bethesda). 2011 6;26(3):192–205. 21670165 10.1152/physiol.00046.2010

[63] Kapoun AM, Liang F, O'Young G, Damm DL, Quon D, White RT, et al B-type natriuretic peptide exerts broad functional opposition to transforming growth factor-beta in primary human cardiac fibroblasts: fibrosis, myofibroblast conversion, proliferation, and inflammation. Circulation research. 2004 3 5;94(4):453–61. 10.1161/01.RES.0000117070.86556.9F 14726474

[64] Lin X, Hanze J, Heese F, Sodmann R, Lang RE. Gene expression of natriuretic peptide receptors in myocardial cells. Circulation research. 1995 10;77(4):750–8. 7554122 10.1161/01.res.77.4.750

[65] Nishikimi T, Maeda N, Matsuoka H. The role of natriuretic peptides in cardioprotection. Cardiovascular research. 2006 2 1;69(2):318–28. 10.1016/j.cardiores.2005.10.001 16289003

[66] Tokudome T, Horio T, Kishimoto I, Soeki T, Mori K, Kawano Y, et al Calcineurin-nuclear factor of activated T cells pathway-dependent cardiac remodeling in mice deficient in guanylyl cyclase A, a receptor for atrial and brain natriuretic peptides. Circulation. 2005 6 14;111(23):3095–104. 10.1161/CIRCULATIONAHA.104.510594 15939815

[67] Tokudome T, Kishimoto I, Horio T, Arai Y, Schwenke DO, Hino J, et al Regulator of G-protein signaling subtype 4 mediates antihypertrophic effect of locally secreted natriuretic peptides in the heart. Circulation. 2008 5 06;117(18):2329–39. 10.1161/CIRCULATIONAHA.107.732990 18443239

[68] Mouli S, Nanayakkara G, AlAlasmari A, Eldoumani H, Fu X, Berlin A, et al The role of frataxin in doxorubicin-mediated cardiac hypertrophy. American journal of physiology Heart and circulatory physiology. 2015 9;309(5):H844–59. 10.1152/ajpheart.00182.2015 26209053

[69] Karagiannis TC, Lin AJ, Ververis K, Chang L, Tang MM, Okabe J, et al Trichostatin A accentuates doxorubicin-induced hypertrophy in cardiac myocytes. Aging. 2010 10;2(10):659–68. 10.18632/aging.100203 20930262 PMC2993796

[70] Lee C, Longo VD. Fasting vs dietary restriction in cellular protection and cancer treatment: from model organisms to patients. Oncogene. 2011 7 28;30(30):3305–16. 10.1038/onc.2011.91 21516129

[71] Lee C, Raffaghello L, Brandhorst S, Safdie FM, Bianchi G, Martin-Montalvo A, et al Fasting cycles retard growth of tumors and sensitize a range of cancer cell types to chemotherapy. Science translational medicine. 2012 3 7;4(124):124ra27 10.1126/scitranslmed.3003293 22323820 PMC3608686

[72] Brunet A, Bonni A, Zigmond MJ, Lin MZ, Juo P, Hu LS, et al Akt promotes cell survival by phosphorylating and inhibiting a Forkhead transcription factor. Cell. 1999 3 19;96(6):857–68. 10102273 10.1016/s0092-8674(00)80595-4

[73] Shim HS, Wei M, Brandhorst S, Longo VD. Starvation promotes REV1 SUMOylation and p53-dependent sensitization of melanoma and breast cancer cells. Cancer research. 2015 3 15;75(6):1056–67. 10.1158/0008-5472.CAN-14-2249 25614517 PMC4359966

