# Correction: Fasting regulates EGR1 and protects from glucose- and dexamethasone-dependent sensitization to chemotherapy

**DOI:** 10.1371/journal.pbio.1002603

**Published:** 2017-05-01

**Authors:** Stefano Di Biase, Hong Seok Shim, Kyung Hwa Kim, Manlio Vinciguerra, Francesca Rappa, Min Wei, Sebastian Brandhorst, Francesco Cappello, Hamed Mirzaei, Changhan Lee, Valter D. Longo

There are errors in the author affiliations. The affiliation “Department of Experimental Biomedicine and Clinical Neuroscience, University of Palermo, Palermo, Italy” was added for Francesca Rappa. The affiliation “Centro Studi Fegato (CSF)-Liver Research Center, Fondazione Italiana Fegato, Trieste, Italy” was removed for Manlio Vinciguerra. The affiliations should appear as shown here.

Stefano Di Biase**^1^**^☯^, Hong Seok Shim**^1^**^☯^, Kyung Hwa Kim**^1^**^☯^, Manlio Vinciguerra**^2,3^**, Francesca Rappa**^5,6^**, Min Wei**^1^**, Sebastian Brandhorst**^1^**, Francesco Cappello**^5,6^**, Hamed Mirzaei**^1^**, Changhan Lee**^1^**, Valter D. Longo^1,7^*

**1** Longevity Institute, Leonard Davis School of Gerontology and Department of Biological Sciences, University of Southern California, Los Angeles, California, United States of America, **2** Institute for Liver and Digestive Health, Royal Free Hospital, University College London (UCL), London, United Kingdom, **3** Center for Translational Medicine (CTM), International Clinical Research Center (ICRC), St. Anne's University Hospital, Brno, Czech Republic, **4** Centro Studi Fegato (CSF)-Liver Research Center, Fondazione Italiana Fegato, Trieste, Italy, **5** Euro-Mediterranean Institute of Science and Technology, Palermo, Italy, **6** Department of Experimental Biomedicine and Clinical Neuroscience, University of Palermo, Palermo, Italy, **7** IFOM, FIRC Institute of Molecular Oncology, Milano, Italy

☯ These authors contributed equally to this work.

*vlongo@usc.edu
