# Supplementary figures and images for: Fasting regulates EGR1 and protects from glucose- and dexamethasone-dependent sensitization to chemotherapy
Source: PLoS Biol. 2017 Mar 30;15(3):e2001951. doi: 10.1371/journal.pbio.2001951 (PMC5373519; doi:10.1371/journal.pbio.2001951)

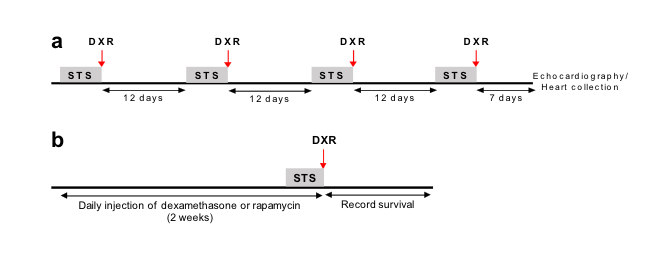

Supplement: S1 Fig — (A) C57BL/6 mice were divided into 4 groups and underwent ad libitum feeding (ad lib), STS (48 h fasting) and/or doxorubicin treatment (DXR, 8 mg/kg). DXR was administrated at the end of each STS cycle. (B) Mice were daily injected with dexamethasone or rapamycin for 2 weeks, before undergoing STS regimen (48h). Insulin group was treated with 1.5 U of insulin twice a day for 2 days before DXR (24 mg/kg) administration at the end of STS. Their survival was monitored for up to 35 days. (TIF) [file pbio.2001951.s001.tif]

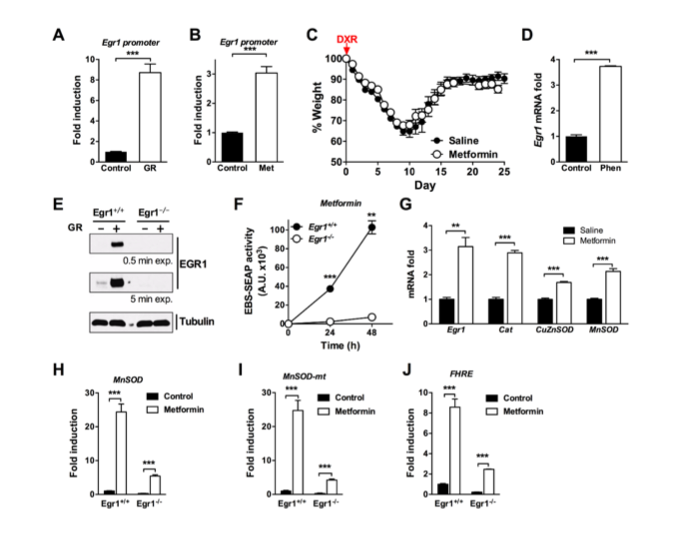

Supplement: S2 Fig — (A, B) H9c2 cardiomyocytes transfected with Egr1-promoter luciferase were treated with GR or metformin (20 mM). Luciferase activity was carried out 6 h post-treatment. (C) Body weight of mice undergoing control and metformin treatment was monitored to exclude body weight loss due to drug administration. Points and bars represent the mean ±s.e.m. (n = 10). (D) H9c2 cardiomyocytes were treated with phenformin (2 mM) for 1 h and Egr1 mRNA levels were measured using qRT-PCR. (E) Egr1+/+ and Egr1-/- MEFs and their response to glucose restriction (GR). (F-J) Egr1+/+ and Egr1-/- MEFs were treated with Met following transient transfection with (G) EGR1-SEAP reporter (SEAP activity was measured and assessed over time by using a chemiluminescent assay), (H-J) MnSOD-luc, MnSOD-mt-luc, or FHRE-luc (luciferase activity was measured 18 h after treatment). P-value <0.05 were considered significant (p-value<0.05, 0.01 and 0.001 are indicated as *, *, and ***, respectively). Underlying data and method of statistical analysis are provided in S1 Data. (TIF) [file pbio.2001951.s002.tif]

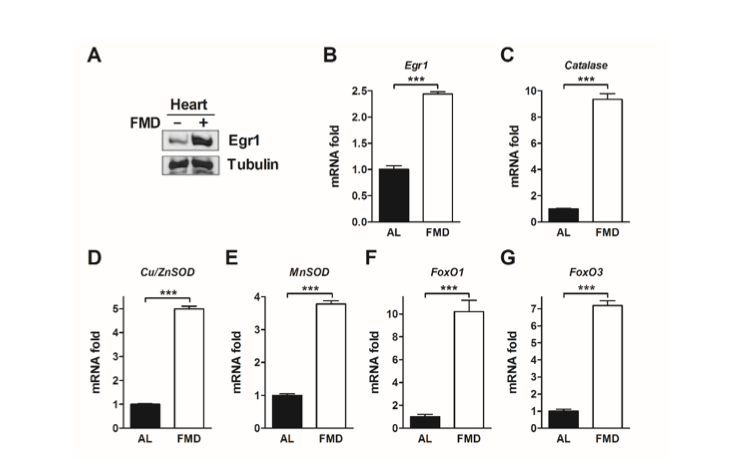

Supplement: S3 Fig — (A-G) Heart tissues from ad lib (AL) and FMD-treated mice were collected and protein and mRNA levels for the indicated genes were assessed by (A) Western blotting and (B-G) qRT-PCR, respectively. P-value <0.05 were considered significant (p-value<0.05, 0.01 and 0.001 are indicated as *, *, and ***, respectively). Underlying data and method of statistical analysis are provided in S1 Data. (TIF) [file pbio.2001951.s003.tif]

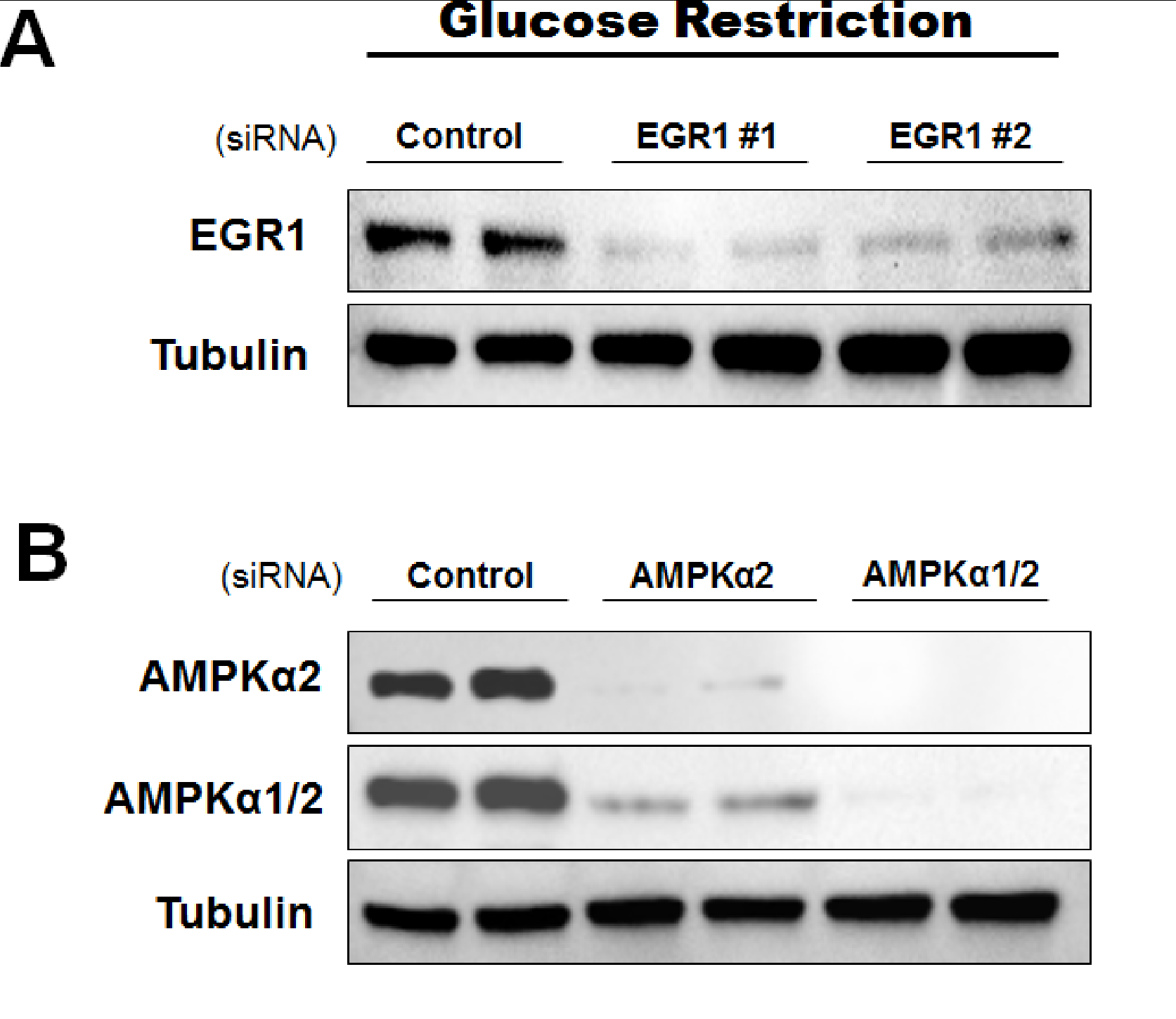

Supplement: S4 Fig — siRNA-mediated knock-down of (A) EGR1 and (B) AMPKα2 and AMPKα1/2 in H9c2 cells following glucose restriction. (TIF) [file pbio.2001951.s004.tif]

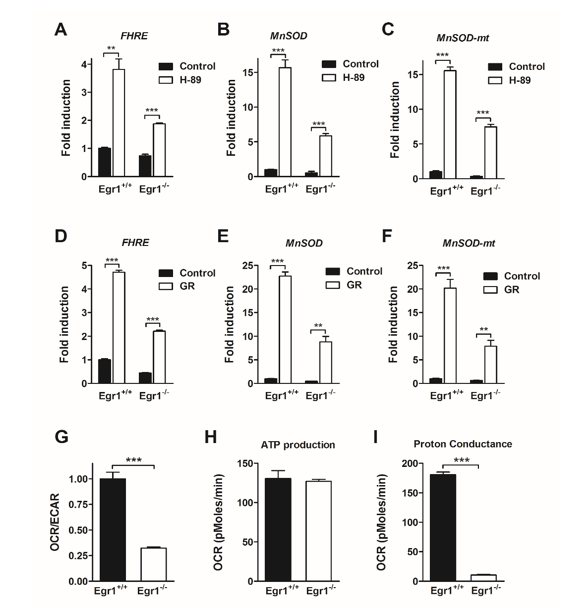

Supplement: S5 Fig — (A-C). Egr1+/+ and Egr1-/- MEFs were transfected with (A) FHRE-luc, (B) MnSOD-mt-luc, or (C) MnSOD-luc, and treated with H89. Luciferase activity was assessed 18 h after treatment. (D-F) The regulation of MnSOD and FOXO responsive genes in Egr1-dependent manner. Egr1+/+ and Egr1-/- MEFs were transfected with (D) FHRE-luc, (E) MnSOD-mt-luc, or (F) MnSOD-luc, and treated with GR medium. Luciferase activity was measured 18 h after treatment. (G-I) EGR1 regulates energy metabolism. (G) Basal levels of OCR to ECAR ratios, (H) ATP production, and (I) proton conductance were measured in Egr1+/+ and Egr1-/- MEFs by the XF96 extracellular flux analyzer. P-value <0.05 were considered significant (p-value<0.05, 0.01 and 0.001 are indicated as *, *, and ***, respectively). Underlying data and method of statistical analysis are provided in S1 Data. (TIF) [file pbio.2001951.s005.tif]
